# Effects of Modulating Actin Dynamics on HER2 Cancer Cell Motility and Metastasis

**DOI:** 10.1038/s41598-018-35284-9

**Published:** 2018-11-22

**Authors:** Sarah Nersesian, Rodette Williams, Daniel Newsted, Kavan Shah, Stephanie Young, P. Andrew Evans, John S. Allingham, Andrew W. Craig

**Affiliations:** 1 Department of Biomedical and Molecular Sciences, Queen’s University, Kingston, Ontario, Canada; 2 Cancer Biology & Genetics division, Queen’s Cancer Research Institute, Kingston, Ontario, Canada; 3 Department of Chemistry, Queen’s University, Kingston, Ontario, Canada

## Abstract

Amplification of HER2 leads to development of HER2-positive (HER2+) cancers with high rates of metastasis compared to other cancer subtypes. The goal of this study was to probe the vulnerability of HER2+ cancer cells to a filamentous actin (F-actin) severing and capping toxin. The growth and viability of human HER2+ breast cancer (HCC1954) and ovarian cancer (SKOV3) cell lines were significantly impaired upon treatment with the marine macrolide mycalolide B (Myc B) at doses above 100 nanomolar. Further testing of Myc B in combination with the antibody-drug conjugate Trastuzumab-emtansine (T-DM1) led to improved killing of SKOV3 cells compared to either treatment alone. At sub-lethal doses, treatment of HER2+ cancer cells with Myc B resulted in rapid loss of leading edge protrusions and formation of aggresomes containing F-actin and the actin regulatory protein Cortactin. This correlated with robust inhibition of HER2+ cancer cell motility and invasion with Myc B treatment. In SKOV3 tumor xenograft assays, intratumoral injections of Myc B impaired HER2+ tumor growth and metastasis, with maximal effects observed in combination with systemic delivery of Trastuzumab. Metastasis of SKOV3 cells to the lungs following tail vein injection was also reduced by Myc B. Together, these findings provide rationale for targeting F-actin in combination with existing therapies for HER2+ cancers to reduce metastasis.

## Introduction

Elevated expression of Human Epidermal Growth Factor Receptor 2 (HER2) due to gene amplification occurs in a subset of cancers with high rates of metastasis^1,2^. High levels of HER2 are detected in breast cancer (20–25%), ovarian cancer (30%), and in several other cancers including gastric, prostate, salivary gland and lung cancers^3–6^. Treatment approaches currently applied to HER2-positive (HER2+) cancers include the small molecule inhibitor Lapatinib, the inhibitory antibody Trastuzumab, and the antibody-drug conjugate Trastuzumab Emtansine (T-DM1)^7–9^. Although these targeted therapies have significantly improved survival rates for HER2+ cancer patients, some tumors develop resistance and progress to metastatic disease^10^. Indeed, therapies that target early steps in the metastatic process may complement existing forms of therapies for HER2+ cancers and improve overall survival rates.

Metastasis involves the dissemination of cancer from the primary tumor to secondary sites, and is the leading cause of cancer-related deaths. To address this, new therapies are needed that target major drivers of metastasis^11,12^. Although T-DM1 allows for targeted delivery of chemotherapy to HER2+ cells, the emtansine warhead disrupts microtubules and therefore largely targets rapidly dividing cancer cells^13^. However, distinct properties of metastasis-initiating cells have been linked to resistance to many existing therapies^14^. Early events in metastasis require rapid extension of specialized cell protrusions that depend on polymerization of filamentous actin (F-actin) to breach basement membranes, invade tissues, and blood vessels or lymphatics^15–17^. Targeting dynamic F-actin in tumor cells may provide additional forms of therapy to limit progression to metastatic disease^18^.

A diverse group of marine macrolide toxins have been identified that disrupt F-actin dynamics^19–21^. Several of these toxins are potent inhibitors of cancer cell growth and survival in studies of cancers cell lines derived from skin, blood, colon, and breast^22–26^. These findings have drawn attention to actin toxins as a potential source of new pharmacological tools and therapeutic agents^27,28^. Indeed, these natural products have inspired the design of potential new cancer drugs targeting F-actin^19,20,29–31^. However, further research is needed to identify candidate toxins, their effects in specific cancer types, and to consider potential modes of delivery to tumor cells^32^.

In this study, we demonstrate that the F-actin severing and capping toxin Myc B induced rapid loss of leading edge protrusions and suppressed motility and invasion of HER2+ breast (HCC1954) and ovarian (SKOV3) cancer cell lines at low nanomolar doses. At slightly higher doses, Myc B was cytotoxic and suppressed cell growth completely. In SKOV3 cells, combination treatments with Myc B and T-DM1 led to increased cytotoxicity compared to either agent alone, and in HER2+ tumor xenograft models, Myc B treatment suppressed both tumor growth and metastasis.

## Results

### Actin toxin Myc B limits growth and survival of HER2+ cancer cell lines

**Figure 1:**
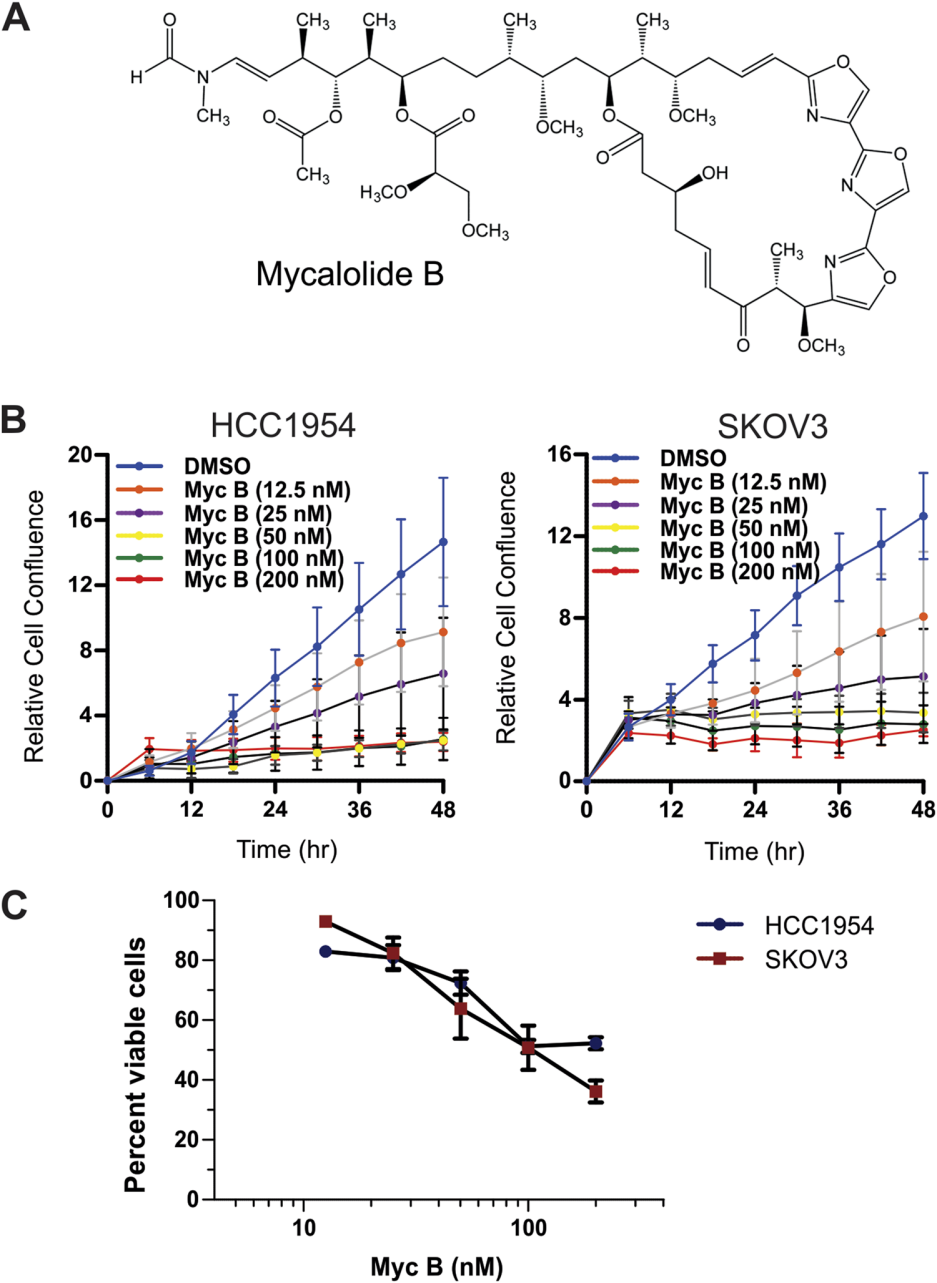
Myc B suppresses growth and viability of HER2+ cancer cells. (**A**) The chemical structure of Myc B is shown. (**B**) Graphs depict the percent change in confluence for SKOV3 cells (left) and HCC1954 cells (right) over 48 hours of treatment with DMSO or Myc B (12.5–200 nM; results for quandruplicate experiments, mean ± SEM) using an IncuCyte Zoom system. (**C**) HCC1954 (breast) and SKOV3 (ovarian) cells were incubated with DMSO (1%) or Myc B (12.5–200 nM) in DMEM supplemented with 2% FBS and PI (1 µM) using an IncuCyte Zoom system for 48 hours. Graphs depict the percentage cell viability as determined at endpoint by measuring total cells and PI positive cells using ImageJ software (results for triplicate experiments, mean ± SEM).

Previous studies have shown that the marine macrolide Myc B (Fig. 1A) targets F-actin via severing and capping mechanisms^33–36^. In this study, we tested the effects of Myc B in HER2+ cancer cells, including HCC1954 breast cancer and SKOV3 ovarian cancer cell lines. With increasing doses of Myc B (0–200 nM), compared to DMSO as a vehicle control, we observed dose dependent inhibition of cell growth over a 48 hour period (Fig. 1B). The effects of Myc B on the viability of both cell lines was assessed by measuring the uptake of propidium iodide (PI) using parallel epiflourescence and phase contrast imaging. Relative to DMSO control treatment that was set at 100% viability, we observed a dose-dependent reduction in cell viability with Myc B treatment, with EC_50_ values of 183 and 105 nM for HCC1954 and SKOV3 cell lines, respectively (Fig. 1C). It is worth noting that low doses of Myc B that had limited cytotoxicity (e.g. 12.5–25 nM) did cause a marked reduction in HER2+ cancer cell growth (see Fig. 1B). Similar results were observed upon Myc B treatment of HER2-negative prostate cancer cell lines (Suppl. Fig. 1). Overall, these results demonstrate that Myc B treatment leads to suppression of cancer cell growth and viability.

### HER2+ cancer cells are sensitive to Myc B and T-DM1 combination treatments

**Figure 2:**
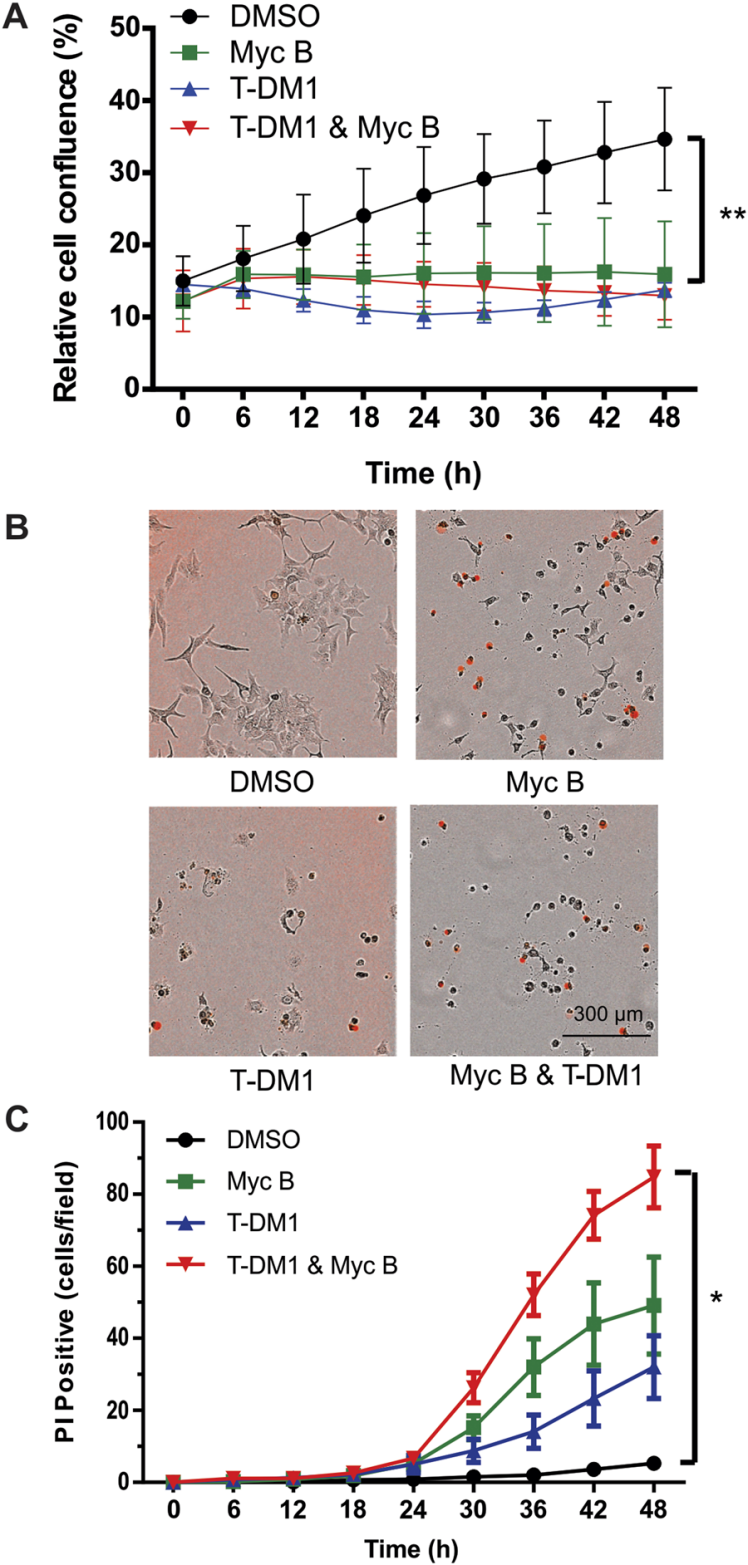
The effects of Myc B and T-DM1 treatments on HER2+ cancer cell growth and cytoxicity. SKOV3 cells were seeded and incubated with DMSO (1%), Myc B (25 nM), T-DM1 (0.5 µg/ml), or a combination of Myc B and T-DM1 in DMEM supplemented with 2% FBS and PI (1 µM) using the IncuCyte Zoom Live Cell Analysis System for 48 hours. (**A**) The graph depicts the percent change in confluence between treatment groups (mean ± SD, a significant difference between DMSO and all other treatment groups is indicated by **P < 0.01). **(B)** Images that are representative of each treatment group at the 48 hour endpoint are shown as merged micrographs of PI fluorescence and phase contrast images of SKOV3 cells. (**C**) Graph depicts quantification of PI positive cells per field for triplicate experiments (mean ± SEM, *P < 0.05).

Considering the importance of T-DM1 in treatment of HER2+ cancers, and the potential for future combination treatments with actin-targeting drugs, we next examined the response of HER2+ cancer cells to combination treatments with Myc B and T-DM1. SKOV3 cells were seeded at low density in media supplemented with submaximal doses of Myc B (25 nM) or T-DM1 (0.5 µg/ml) alone, or in combination. The cell confluence increased significantly in DMSO group compared to treatments with Myc B, T-DM1, or the combination (Fig. 2A). To distinguish between reduced cell growth and cytotoxicity, we visualized PI uptake in each treatment group at endpoint. Compared to DMSO controls, treatment with Myc B and/or T-DM1 led to increased PI uptake in SKOV3 cells within 48 hours (Fig. 2B). Quantification of these results demonstrated that the combination of Myc B and T-DM1 was most effective in promoting killing of SKOV3 cells (Fig. 2C). Together, these results demonstrate that actin-disrupting agents like Myc B are compatible with killing of HER2+ cancer cells with the targeted clinical grade inhibitor T-DM1.

### Loss of leading edge protrusions in HER2+ cancer cells treated with Myc B

To evaluate the extent of F-actin disruption upon Myc B treatment of HER2+ cancer cells, we measured total F-actin levels in SKOV3 cells treated with DMSO or Myc B (200 nM) for 4 hours. Cells were permeabilized, and stained with FITC-Phalloidin prior to analysis by flow cytometry. As expected, Myc B treatment triggered a significant reduction in the overall F-actin content of SKOV3 cells (Fig. 2A**)**. To visualize the location and kinetics of F-actin disruption by Myc B treatment, we established SKOV3 cells that stably express the F-actin reporter LifeAct-GFP, and performed live cell imaging using a super-resolution confocal microscopy platform. At a sublethal dose of Myc B (25 nM), cortical F-actin and membrane projections began to collapse within minutes, and large F-actin aggregates began to form (Suppl. Fig. 2, Video 1). Similar effects were observed using spinning disk confocal microscopy for SKOV3-LifeAct-GFP cells treated with 50 nM dose of Myc B, with complete collapse of F-actin-rich protrusions leading to accumulation of F-actin aggregates within one hour of treatment (Fig. 2B, Video 2). Importantly, these effects were not observed in media supplemented with only the vehicle DMSO (Fig. 2B, Video 3).

**Figure 3:**
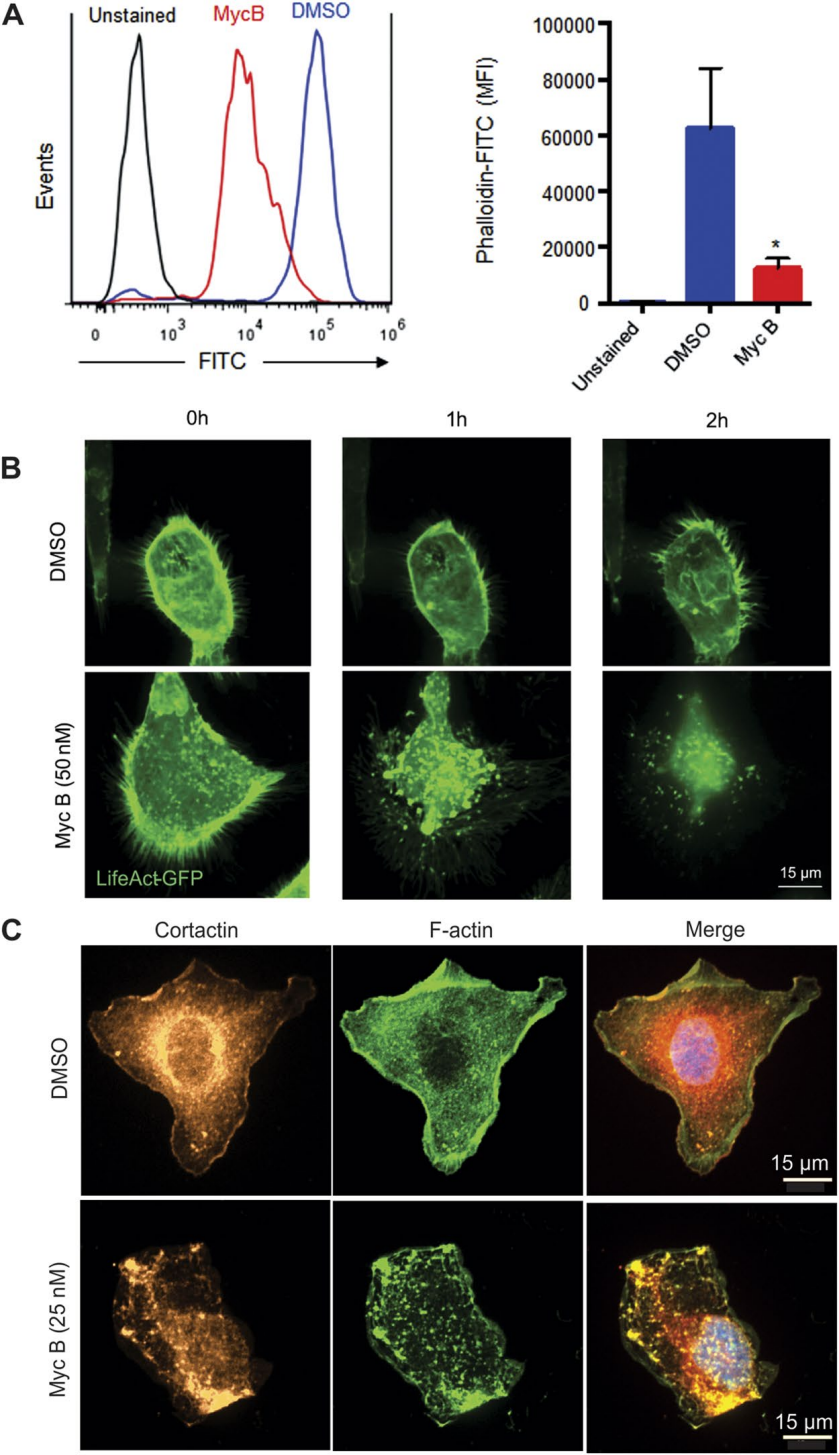
Dynamics of F-actin disruption following Myc B treatment of HER2+ cancer cells. (**A**) A representative flow cytometry histogram (left) is shown for SKOV3 cells treated with DMSO (1%) or Myc B (200 nM) for 4 hours prior to permeabilization and staining with FITC-Phalloidin. Graph depicts FITC-Phalloidin median fluorescence intensity (MFI) for 4 experiments (mean ± SEM, *P < 0.05). (**B**) Representative confocal micrographs are shown for selected time points of live cell imaging for SKOV3 cells expressing LifeAct-GFP (green) treated with either DMSO (1%) or Myc B (50 nM; scale bar indicates 15 µm). **(C)** Representative confocal micrographs are shown for SKOV3 cells treated with DMSO (1%) or Myc B (25 nM) for 2 hours prior to immunostaining with anti-Cortactin (red) along with counterstaining of F-actin (FITC-Phalloidin, green) and nuclei with DAPI (blue).

To test the effects of Myc B on actin regulatory proteins, we visualized the changes in localization of the F-actin binding protein Cortactin that functions in cancer cell motility and invasion. SKOV3 cells were treated with DMSO or Myc B (25 nM) for 2 hours, and the subcellular localization of endogenous Cortactin and F-actin were analyzed by immunoflourescence staining followed by confocal microscopy. In DMSO control treatment, cells with a polarized and motile phenotype were observed with leading edge protrusions staining positive for Cortactin and F-actin (Fig. 3C). Conversely, in Myc B treated cells, Cortactin was localized in large puncta that also contained F-actin, consistent with formation of aggresomes (Fig. 3C)^37^. Together, these results demonstrate that Myc B exposure leads to rapid collapse of actin-based cell protrusions in HER2+ cancer cells, which is expected to severely disrupt cancer cell motility.

### Myc B suppresses HER2+ cancer cell migration and invasion

**Figure 4:**
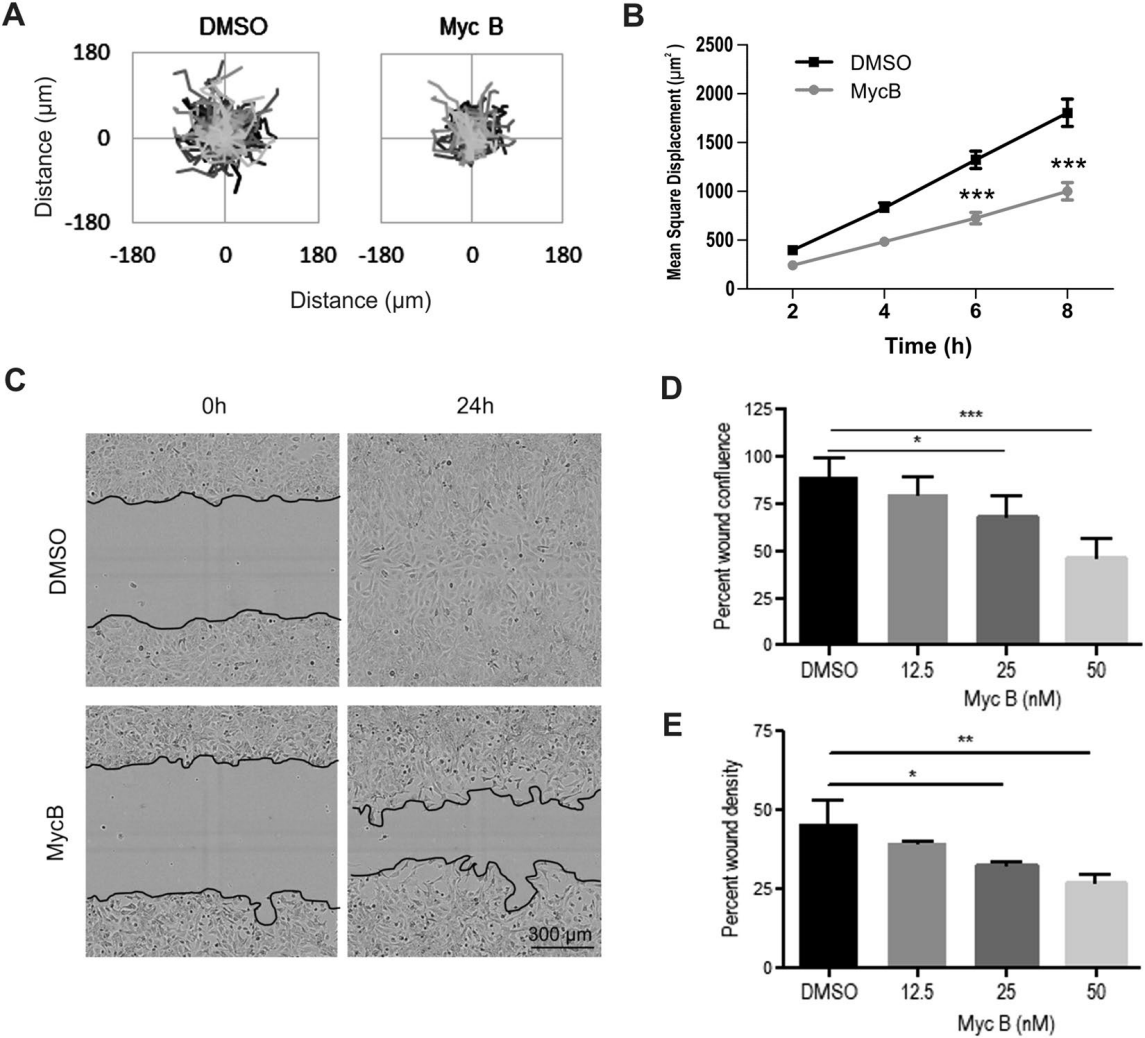
Mycalolide B inhibits HER2+ cancer cell motility. (**A**) Cell motility spider plots are shown for SKOV3 cells treated with either DMSO (1%) or Myc B (25 nM) for 12 hours (n = 255 cells for tracking). (**B**) Graph depicts the mean square displacement for each treatment group (mean ± SEM, ***P < 0.001). (**C,D**) SKOV3 cells were treated with DMSO or Myc B (12.5–50 nM) in scratch wound migration and invasion assays, as described in Materials and Methods. Representative phase contrast micrographs (**C**) are shown for migration assays at time 0 and 24 hours (black lines indicate the boundary of the wound area; Myc B dose of 50 nM). (**D,E**) Graphs depict the percentage of the wound area confluence after 24 hours of the indicated treatments for migration assays (**D**), and percent wound density for invasion assays (**E**) that were conducted in triplicate (mean ± SEM, *P < 0.05, **P < 0.01, ***P < 0.001).

Since sublethal doses of Myc B disrupt leading edge protrusions in HER2+ cancer cells, we tested the effects of Myc B on random and directional cell motility, as well as cell invasion through collagen-rich extracellular matrix. To measure random cell motility, we tracked movements of >250 individual SKOV3 cells treated with either DMSO or Myc B (25 nM) for 12 hours. The spider plots, with lines representing the movement of each cell, demonstrated that Myc B treatment led to impaired random cell motility (Fig. 4A). Quantification of the overall mean square displacement for each treatment revealed a ~50% reduction in motility with this sublethal dose of Myc B (Fig. 4B). To evaluate effects of Myc B on directional cell migration, a scratch wound assay was performed using SKOV3 cells. In this assay, SKOV3 cells treated with vehicle were capable of completely covering the wound area within 24 hours, whereas treatment with Myc B led to reduced wound closure (Fig. 4C), and in a dose-dependent manner (Fig. 4D). Similar results were observed for invasion assays wherein the wound area was overlayed with Matrigel, with Myc B showing dose dependent suppression of HER2+ cancer cell invasion (Fig. 4E, Videos 4 and 5). Similar dose-dependent suppression of cell motility was observed in HER2-negative prostate cancer cells treated with Myc B (Suppl. Fig. 3). Together, these results suggest that many metastatic cancer types may benefit from treatment with Myc B or related inhibitors.

**Figure 5:**
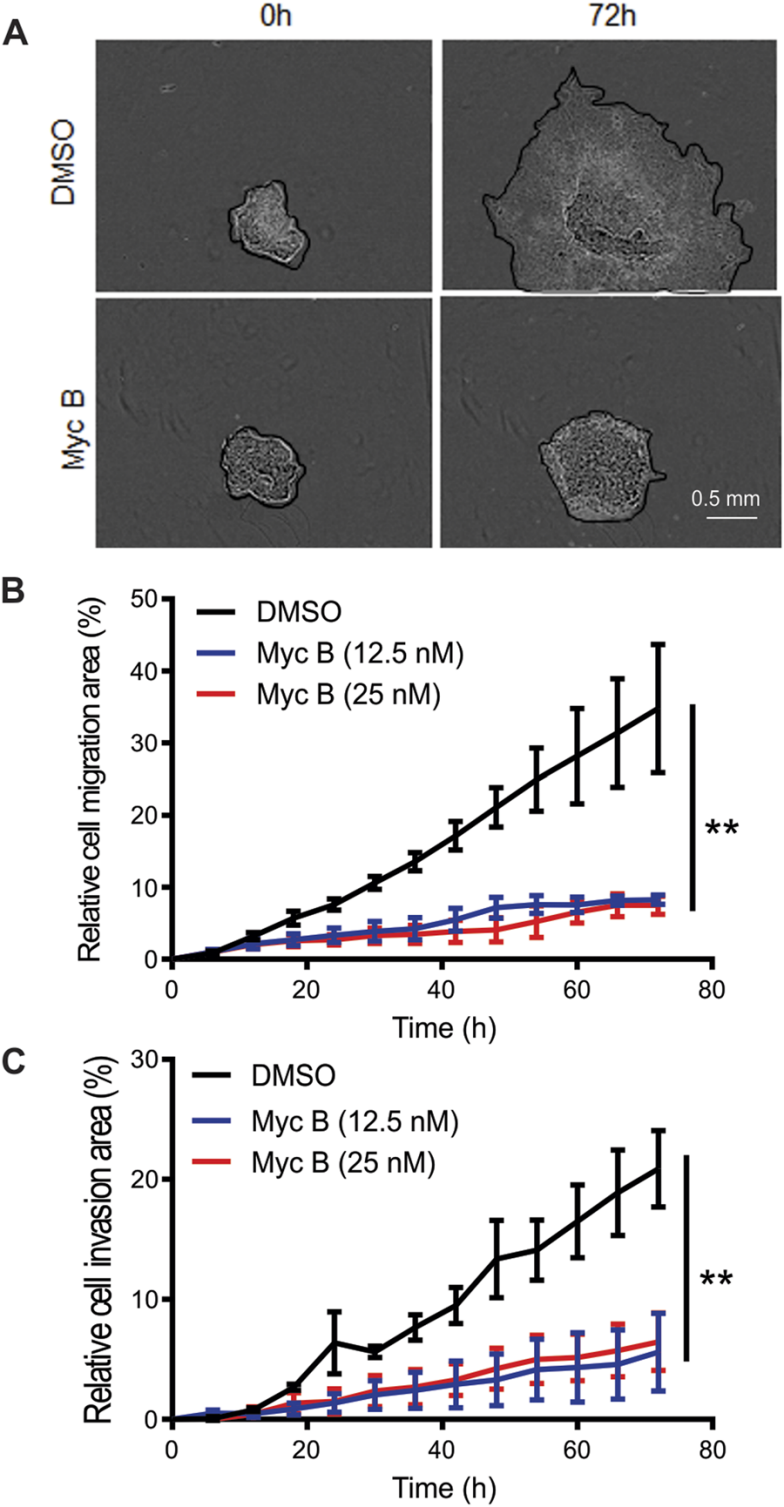
Myc B inhibits migration and invasion of HER2+ spheroids following reattachment. SKOV3 cell spheroids were grown in low adhesion wells then moved to adhesive wells to reattach during treatment in media supplemented with DMSO (1%) or Myc B (12.5 or 25 nM) and imaged over 72 hours using an IncuCyte ZOOM, as described in Materials and Methods. (**A**) Representative phase contrast images of SKOV3 cells treated with DMSO or Myc B (25 nM) in the spheroid migration assays at time 0 and at 72 hours post attachment. (**B,C**) Graphs depict quantification of percent change in area of cell migration (**B**) or cell invasion through Matrigel (**C**). Significant differences were observed between DMSO and Myc B treatments were observed at endpoint for triplicate experiments (mean ± SEM, **P < 0.01).

To extend these studies to a 3D model of cell motility and invasion, SKOV3-LifeAct-GFP cells were grown as spheroids for 3 days in wells under low adhesion conditions. Spheroids were then transfered to wells permissive for cell adhesion and subsequent radial migration or invasion through Matrigel was measured in the presence of DMSO or Myc B (12.5 or 25 nM). Phase contrast imaging of the spheroids over 72 hours revealed that sublethal doses of Myc B significantly impaired 3D migration of SKOV3 cells (Fig. 5A,B). In parallel assays, spheroids were embedded in Matrigel to measure 3D invasion over 72 hours. Quantification of these results also revealed significantly reduced SKOV3 cell invasion with Myc B treatment (Fig. 5C). These results highlight the efficacy of Myc B to limit the motile and invasive properties of HER2+ cancer cells in a 3D model.

### Myc B suppresses HER2+ tumor growth and metastasis and complements effects of Trastuzumab

**Figure 6:**
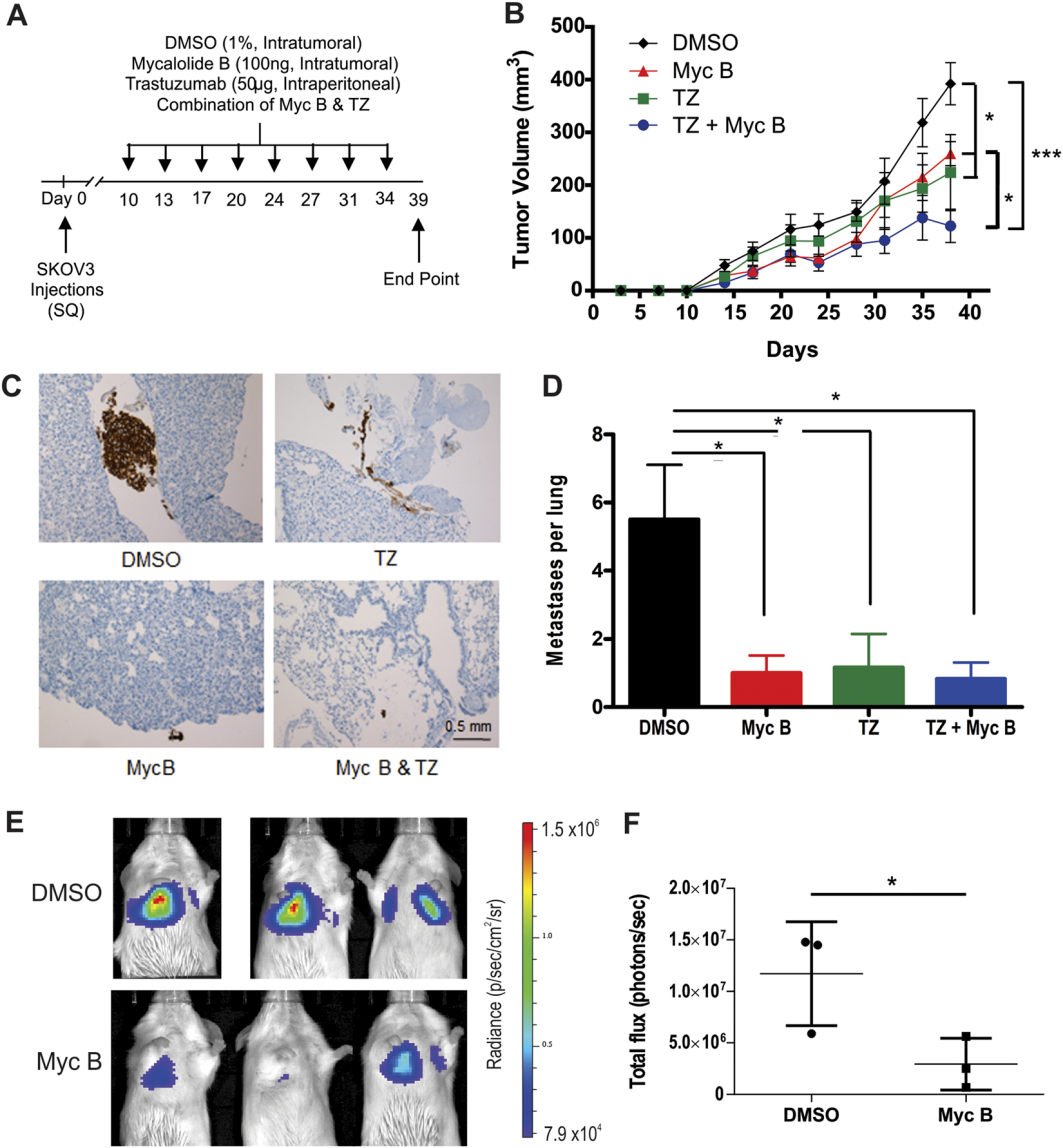
Combination treatments with Myc B and Trastuzumab suppresses HER2+ tumor growth and metastasis in a tumor xenograft model. (**A**) A timeline and treatment schedule is presented for subcutaneous tumor xenograft studies using SKOV3 cells injected in female Rag2^−/−^ IL2γc^−/−^ mice and treated on the indicated days with either intratumoral injections of DMSO or Myc B alone, Trastuzumab (TZ) alone, or the combination of TZ and Myc B (n = 6/group). **(B)** Graph depicts the tumor volumes for each treatment group over the 39 day period (mean ± SEM, 2-way ANOVA with Tukey’s multiple comparison testing, *P < 0.05, ***P < 0.001). **(C)** To visualize lung metastases, lung tissue sections were subjected to immunohistochemistry staining of HER2, and representative micrographs are shown for each treatment group (scale bar indicates 0.5 mm). **(D**) Graph depicts quantification of micrometastases per lung section (n = 6/group, mean ± SEM, *P < 0.05). **(E)** SKOV3-Luc cells with vehicle (saline/1% DMSO) or Myc B (saline/100 ng Myc B) were injected by tail vein in female Rag2^−/−^ IL2γc^−/−^ mice (n = 3 mice/group), and after 7 days the lung seeding efficiency was measured by bioluminescence imaging of D-luciferin-injected mice. **(F)** Graph depicts the total photon flux between DMSO and Myc B treatment groups (mean ± SD, *P < 0.05).

To extend our studies to a metastatic HER2+ tumor model, we established subcutaneous SKOV3 tumors in female Rag2^−/−^:IL2γc^−/−^ mice (lacking NK, B and T cells). When palpable tumors were detected (day 10–14), mice were randomized between the following treatments every 2–3 days: intra-tumoral injections of DMSO (1% in saline) or Myc B (100 ng), or with intraperitoneal injections of Trastuzumab alone (TZ, 50 µg), or in combination with Myc B (Fig. 6A). Using calipers to measure tumor volumes, we observed reduced tumor growth for Myc B and TZ treatment groups (Fig. 6B). The combination treatment group showed significant reduction in tumor growth at endpoint with DMSO control and Myc B treatment alone (Fig. 6B). It is worth noting that no differences in body weight were observed between treatment groups (Suppl. Fig. 4A), suggesting that this dose and delivery of Myc B was not overtly toxic. At endpoint, the isolated tumors were significantly smaller in the Myc B, TZ and combination treatment groups (Suppl. Fig. 4B). However, no overt differences in tumor tissue histology were noted between treatment groups (Suppl. Fig. 4C). To test the effects of Myc B on F-actin levels in the tumors, we prepared cryosections for staining of F-actin (Phalloidin) and the tumor nuclei (DAPI). The Myc B treated tumors had less density of F-actin compared to DMSO treated control tumors (Suppl. Fig. 4D,E).

To test for effects of Myc B treatments on metastasis-initiating cells, we examined lung tissues from the above xenograft study for metastatic HER2+ tumor cells by immunohistochemistry staining with an antibody specific for human HER2. In the DMSO control cohort, we observed several large metastases in the lung tissue, and these large metastases (>50 cells) were not detected in Myc B or TZ treatment groups (Fig. 6C). In fact, only a small number of HER2+ micrometastases (<10 cells) were detected in TZ or Myc B treatment groups (Fig. 6C). Quantification of these results demonstrated robust suppression of metastasis with Myc B, TZ, or combination treatments (Fig. 6D).

To directly address the effects of Myc B on HER2+ cancer metastasis independent of effects on tumor growth, we performed tail vein injections of SKOV3-Luc cells mixed with either Saline/DMSO control or Saline/Myc B in female Rag2^−/−^:IL2γc^−/−^ mice. After 7 days, the lung seeding efficiency was measured using biophotonic imaging of D-luciferin-injected mice. Compared to DMSO control mice with high levels of bioluminescence detected within the lungs, the Myc B treated group showed only modest lung seeding efficiency (Fig. 6E). Quantification of these results showed that Myc B treatment leads to a significant reduction in total photon flux (Fig. 6F). Together, these results provide compelling evidence that Myc B has anti-metastasis effects in HER2 tumor models, and is compatible with use of clinical grade inhibitor TZ.

## Discussion

Metastasis remains the leading cause of cancer related deaths worldwide^20^, and is poorly controlled by current therapies^29^. This study tested the effects of interfering with F-actin dynamics in HER2+ cancer cells, in combination with existing HER2+ cancer therapies, as an alternative approach to target key properties of metastasis-initiating cells^11,16,17,29^. Here, we demonstrate that Myc B is a potent inhibitor of HER2+ breast and ovarian cancer cell growth and viability. Myc B was also effective in combination with T-DM1 for improved killing of SKOV3 cells *in vitro*. At sublethal doses, Myc B treatment also impaired HER2+ cancer cell migration and invasion, and these effects correlated with rapid loss of leading edge protrusions in HER2+ cancer cells. These effects of Myc B were not exclusive to HER2+ cancer cells, as we observed similar inhibitory effects on cell viability and motility in two prostate cancer cell lines. This builds on a series of studies of Myc B and other actin toxins in other cancer types^22,23,25,26,35,38,39^, however, few studies have tested the effects of actin toxins on tumor progression and metastasis *in vivo*. Here, we show that Myc B can slow tumor growth and dramatically suppress metastasis in a HER2+ tumor xenograft models of spontaneous or experimental lung metastasis. Importantly, Myc B treatments were also effective in combination with the clinical grade inhibitor Trastuzumab in the tumor xenograft model.

It is clear from our live cell imaging experiments that Myc B treatment causes rapid collapse of filopodia and lamellipodia at the cell cortex leading to formation of aggresomes. At later times, these aggresomes appear to be trafficked within Myc B treated cells, which may allow degradation or recycling of the actin subunits and actin binding proteins. Indeed, aggresomes induced by jasplakinolide were associated with, and trafficked on, microtubules with dissolution by lysosomes and autophagosomes^37^. With the collapse of both filopodia and lamellipodia following Myc B treatment, this suggests that both bundled and cross-linked networks of F-actin are disrupted via the actin severing activity of Myc B. Another target of Myc B is Dynactin, a complex containing actin and Actin-related protein-1 (Arp1) subunits involved in retrograde transport of neuronal vesicles^34^. Dynactin also participates in vesicular trafficking of matrix metalloproteinase-14 (MT1-MMP or MMP-14)^40^. Given the involvement of MMP-14 in HER2+ cancer cell invasion^41–43^, it will be interesting to test for the effects of Myc B on Dynactin and MMP-14 activity in these models in future studies aimed at understanding comprehensive effects of actin disruption in metastatic cancers.

Previous studies of the actin toxins Latrunculin A and Chondramide have shown promising results in tumor xenograft models, with no evidence of acute toxicities^31,44^. Along with our study, these results suggest that targeting the augmented actin polymerization dependence in metastatic cancer cells may lead to new treatments targeting metastasis. Further study of Chondramide effects on tumor-associated macrophages (TAMs) revealed interesting differences in cytotoxicity and cytokine production amongst TAM subtypes^45^. The tumor suppressive effects of M1 TAMs were augmented by Chondramide via activation of SAPK/JNK and NFκB pathways and elevated TNFα production^45^. Although Chondramide and Myc B are mechanistically distinct (G-actin nucleation inhibitor vs. F-actin severing), it will be interesting to test for changes in TAM content or phenotype in the Myc B treatment groups in our SKOV3 tumor xenograft assays. Further testing in immune competent models will also be critical to address potential unwanted side effects of actin toxins on other motile immune cells implicated in tumor surveillance or killing.

With the identification of receptors that are specific or enriched within metastatic cancers, new tools and targeted therapies can be developed^3,46^. Although clinical grade antibodies like Trastuzumab have made an impact, the increased potency of T-DM1 has improved response rates in some metastatic HER2+ cancers. However, we expect that the repertoire of antibody drug conjugates will require further expansion to provide more diversity in their mode of actions. We predict that development of novel antibody-drug conjugates, or nanoparticle carriers, with F-actin-disrupting payloads (synthetic derivatives of Myc B) will improve targeting of metastatic cancers and improve outcomes for patients with advanced cancers. T-DM1 has already improved care of patients with HER2+ breast cancers that have progressed or relapsed on standard therapies^9,10^. In this study, we observed benefits of combining the actin toxin Myc B with T-DM1 to promote killing of SKOV3 cells *in vitro*. Our tumor xenograft studies also suggest that Myc B was compatible with Trastuzumab-based therapy that limited tumor growth *in vivo*. The treatments with Myc B or Trastuzumab also suppressed metastasis in our HER2+ tumor models. This is consistent with HER2 signaling-driven cell motility being sensitive to Trastuzumab^47^, and the actin cytoskeleton reorganization being sensitive to Myc B (this study). Overall, combination therapies targeting both HER2 and actin polymerization may allow for comprehensive elimination of HER2+ tumors and metastases.

In conclusion, this study identifies a vulnerability in metastatic HER2+ cancers to the disruption of actin polymerization by an actin toxin. The delivery of this toxin to HER2+ cancer cells or xenograft tumors in future formulations with antibody-drug conjugates or nanoparticles may lead to improved treatments for metastatic cancer. These findings provide rationale to develop and test new actin toxin-based therapies that complement existing therapies for HER2+ and possibly metastatic other cancers.

## Materials and Methods

### Proteins, Reagents and Cell Culture

Myc B was purchased from Wako Chemicals (USA) and stored at −20 °C in DMSO. Trastuzumab (TZ) and T-DM1 were provided by Genentech. HEK293T, SKOV3 and HCC1954 cell lines were obtained from American Type Culture Collection, and authenticated by STR profiling. All cell lines were maintained in Dulbecco Modified Eagle Medium (DMEM, Multicell) supplemented with 10% fetal bovine serum (FBS, Multicell) and 1% antibiotics-antimycotic (Multicell), and cultured in a humidified incubator at 37 °C with 5% CO_2_.

### Propidium Iodide Cytotoxicity Assays

Cytotoxicity was measured via uptake of propidium iodide (PI) from supplemented growth medium using an IncuCyte ZOOM Live-Cell Analysis System (Essen BioScience). Cells (2 × 10^4^) were seeded in triplicate in a 96-well plate, and media with 1 µM PI (Biotium) and 2% FBS was added 24 hours later. Following addition of DMSO or Myc B at the indicated doses, the plate was placed in the IncuCyte ZOOM system. After 48 hours, the numbers of PI positive cells and cell confluence were determined using IncuCyte Software. Total cell counts were determined using ImageJ software. The median effective dose (ED_50_) was calculated by non-linear regression using GraphPad Prism software.

### Flow cytometry

The effects of Myc B on F-actin content of HER2+ cancer cells was quantified by flow cytometry analysis of permeabilized cells labeled with FITC-Phalloidin. HCC1954 and SKOV3 cells (3 × 10^6^) were seeded in a 12-well plate, and 24 hours later treated with Myc B (200 nM) for 4 hours. Cells were collected and fixed using 4% paraformaldehyde for 15 minutes at room temperature, permeabilized using 0.2% Triton X-100 for 10 minutes, and incubated with FITC-Phalloidin (1:200, Sigma) for 1 hour at 4 °C. Cells were washed in PBS and analyzed using a FC500 Series Beckman Coulter flow cytometer, with analysis performed using FloJo software.

### Life-Act-GFP expression and live cell imaging

A lentivirus expressing Life-Act-GFP was used to transduce HCC1954 and SKOV3 cells to visualize the F-actin dynamics in live cells. For viral production, HEK293T cells (5 × 10^5^) were plated in 6-well plate coated with 0.01 mg/m Poly L Lysine (Electron Microscopy Sciences). 24 hours later, cells were co-transfected with pLenti.PGK.LifeAct-GFP (Addgene, cat#51010), PAX2 and MD2G plasmids using X-tremeGENE HP DNA Transfection Reagent (Roche Life Sciences) in serum free DMEM. Filtered virus was then collected and added to adherent HCC1954 and SKOV3 cells (5 × 10^4^) in a 6-well plate. To visualize F-actin dynamics in real time at high resolution detail SKOV3-LifeAct-GFP cells (6 × 10^4^) were seeded on #1.5 glass coverslips coated with 10 µg/ml human fibronectin in live-cell chamber. Cells were imaged at baseline and for 4 hours following treatment with Myc B (25 nM) using a LSM800 Zeiss Laser Scanning Confocal with super-resolution detector Airyscan (100X objective; Carl Zeiss Canada).

### Immunofluorescence staining

Immunofluorescence was used to visualize the effects of Myc B treatment on specific F-actin structures. Acid washed coverslips were coated with human fibronectin prior to seeding of SKOV3 cells, and treatment with DMSO or Myc B (25 nM) for 2 hours. Cells were then fixed with 4% PFA, permeabilized in 0.2% Triton X-100 followed by overnight incubation with anti-cortactin (1:200, Millipore ab#3852) antibody in a humidity chamber at 4 °C. Coverslips were then rinsed with PBS and incubated in the dark for 1 hour at RT with Alexa Fluor® 568-conjugated goat anti-rabbit IgG (1:2000, Invitrogen), TRITC-Phalloidin (1:200, Sigma), and DAPI (1:400, Sigma). Images were acquired using a Quorum WaveFX-X1 spinning disc confocal system (Quorum Technologies Inc., Guelph), and analyzed using Metamorph software.

### Random cell motility tracking

The effects of Myc B on motility of HER2+ cancer cells was evaluated using real-time tracking of individual cells at low confluence in regular growth media. Briefly, cells (2 × 10^4^) were seeded in triplicate in a 96-well plate, and were treated with Myc B (25 nM) the next day, and placed in the IncuCyte ZOOM system. Images were captured every 2 hours for a period of 12 hour period using a 10x objective. Images were then exported, aligned, and individual cells tracked using the Image J MTrack2 plugin to assign X-Y coordinates at each time point. DiPer software was used to create spider plots and determine mean square displacement over time for 255 cells/condition.

### Directional cell migration and invasion assays

To quantify directional cell migration SKOV3 cells (2.5 × 10^4^) were seeded in triplicate in a 96-well ImageLock plate (Essen BioScience). At approximately 90% confluence a scratch was induced using the IncuCyte Woundmaker (a 96-well wound making tool, Essen BioScience). Following wounding, and removal of non-adherent cells, media was added containing the indicated doses of Myc B. The plate was then inserted into the IncuCyte Zoom Live Cell Analysis System and imaged every 2–3 hours for 24 hours. Percentage of wound closure was determined through IncuCyte ZOOM Scratch Wound Analysis. Invasion assays were performed in parallel, with overlay of 5% Matrigel following creation of the wound, and addition of the indicated doses of Myc B. Percentage of wound closure was determined through IncuCyte ZOOM Scratch Wound Analysis.

### Spheroid migration and invasion assays

To evaluate effects of Myc B on 3D HER2+ cancer cell migration and invasion assays, we grew these cells as spheroids prior to treatment and measuring cell migration and invasion. Briefly, a 96-well round bottom plate was coated with poly(2-hydroxymehtyl methacrylate) for 48 hours to create low adhesive conditions. SKOV3 cells (1 × 10^3^) were seeded and formed spheroids within 72 hours. Spheroids were carefully removed using a 100 µl pipette tip and placed in 96-well round bottom plate in media with or without Myc B (25 nM). For invasion assays, media also included 5% Matrigel (BD Biosciences). After 72 hours of phase contrast imaging using an IncuCyte ZOOM system, the radial migration or invasion of SKOV3 cells was measured using IncuCyte ZOOM software to define the area of migration or invasion.

### Tumor xenograft assays and tissue staining

All animals were housed in a specific pathogen-free facility and procedures were approved by the Queen’s University Animal Care Committee in accordance with the Canadian Council on Animal Care guidelines. To evaluate effects of Myc B and Trastuzumab on tumor progression, subcutaneous injections of SKOV3 cells (5 × 10^6^) were performed in female Rag2^−/−^ IL2γc^−/−^ mice. Following detection of palpable tumors on day 14, mice were treated every 2–3 days with either intra-tumoral injection of vehicle (PBS/1% DMSO) or Myc B (100 ng) alone, or intraperitoneal injection of Trastuzumab (50 µg), or the combination of Trastuzumab and Myc B injections as described above. Body weights and tumor volumes were measured every 2–3 days until the endpoint was reached (tumor length >1.7 cm). Tumor volumes were calculated using the equation TV = 0.5 × (length × width^2^), with length and width measurements obtained using calipers. Whole lungs were formalin fixed and paraffin embedded. Sections were prepared and stained with anti-HER2 using the Discovery XT Staining System (Ventana Medical Systems, Inc.). Antigens were retrieved with an EDTA pH 8.0 solution and slides incubated with rabbit anti-HER2 (1:100) antibody (Roche). HER2 immunohistochemistrry (IHC) staining was visualized using DAB with haematoxylin counterstaining (Queen’s Laboratory for Molecular Pathology). Lung metastasis were scored as micro-metastases. Although larger metastases (>50 cells) were observed in the lungs of vehicle controls, these metastases were also scored as a single micro-metastasis since they likely originated from a single tumor cell. Primary tumor tissues were processed for cryosectioning, with 20-μm sections post-fixed in acetone, blocked for 1 hour with 3% bovine serum albumin, and stained with Alexa555-conjugated Phalloidin (1:400, Cytoskeleton Inc.) and DAPI (1:400, Sigma-Aldrich) for 1 hour at room temperature. Images were acquired using an Olympus BX51 epifluorescence microscope equipped with a Q Color5 digital camera (20X objective; images were acquired using QCapturePro software). The relative intensities of DAPI and Phalloidin staining were quantified using Image J software (NIH), and relative Phalloidin intensity (ratio Phalloidin signal/DAPI signal) reported for each field (n = 8–10 fields/mouse). Experimental metastasis assays were conducted using SKOV3-Luc cells (10^6^) in growth media (0.1 ml) mixed with PBS/1% DMSO (0.1 ml) or PBS/Myc B (100 ng in 0.1 ml) on ice. The cell suspensions (0.2 ml) were injected into the tail vein of female Rag2^−/−^ IL2γc^−/−^ mice. After 1 week, mice were injected i.p. with D-luciferin (0.2 ml of 15 mg/ml stock) and anesthetized with isoflurane. After 10 minutes, mice were placed in a biophotonic imaging chamber to acquire bioluminescence and brightfield images (IVIS Lumina LT Series III, Perkin Elmer). The radiance and total flux were calculated using Living Image software (Perkin Elmer).

### Statistical analysis

Unless indicated otherwise, all experiments were performed in triplicate and presented as mean +/− standard error (SEM). One-way or two-way ANOVA with Tukey’s multiple comparison testing were used to compare across treatment groups and/or times, with statistical significance defined as *P* < 0.05 (GraphPad Prism).

## Electronic supplementary material

Supplementary Figures

Suppl Video 1

Suppl Video 2

Suppl Video 3

Suppl Video 4

Suppl Video 5
