# Supplementary material for: Effects of Modulating Actin Dynamics on HER2 Cancer Cell Motility and Metastasis
Source: Sci Rep. 2018 Nov 22;8:17243. doi: 10.1038/s41598-018-35284-9 (PMC6250728; doi:10.1038/s41598-018-35284-9)
Supplement: Supplementary file 1 — Supplementary Figures [file 41598_2018_35284_MOESM1_ESM.pdf]

Effects of Modulating Actin Dynamics on HER2 Cancer Cell Motility and Metastasis

Sarah Nersesian[1,2], Rodette Williams[1,2], Daniel Newsted[1,2], Kavan Shah[1,2], Stephanie Young[1,2], P. Andrew Evans,[3] John S. Allingham[1] and Andrew W. Craig[1,2]

[1]Department of Biomedical and Molecular Sciences, Queen's University, Kingston, Ontario, Canada; [2]Cancer Biology & Genetics division, Queen's Cancer Research Institute, Kingston, Ontario, Canada; [3]Department of Chemistry, Queen's University, Kingston, Ontario, Canada

Supplementary Information

Suppl. Figure 1. Myc B suppresses growth and viability of HER2-negative cancer cells

Suppl. Figure 2. Kinetics of F-actin disruption by Myc B in live SKOV3-LifeAct cells

Suppl. Figure 3. Myc B treatment suppresses HER2-negative cancer cell motility

Suppl. Figure 4. Representative phenotype of SKOV3 subcutaneous xenograft tumor sections.

Supplementary Videos 1-5.

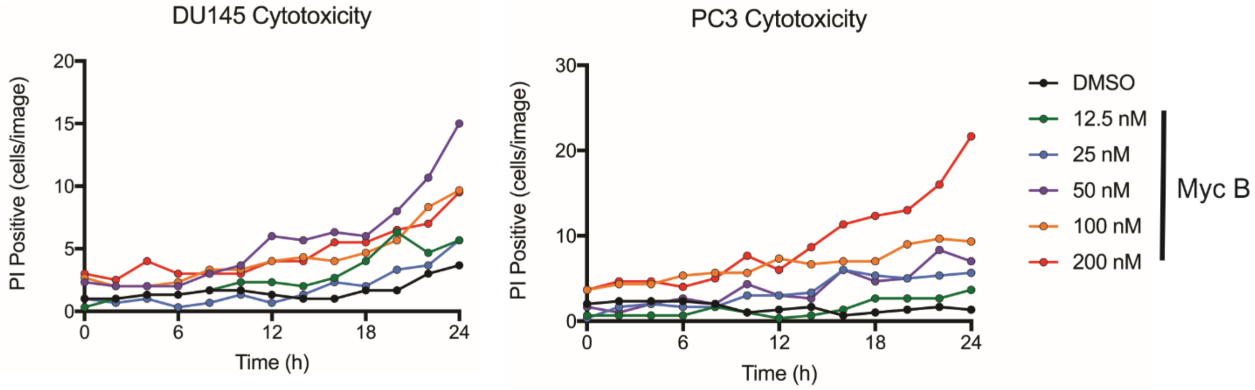

Suppl. Fig. 1. Myc B suppresses growth and viability of HER2-negative cancer cells. Prostate cancer PC3 and DU145 cells (2 x10^3) were seeded in triplicate in a 96-well plate. Media with 1 µM propidium iodide (PI) and 2% FBS was added 24 hours later. Following addition of DMSO (1%) or Myc B (12.5 – 200 nM), the plate was then placed in the IncuCyte ZOOM system for 24 hours, with images acquired every 2 hours. Graphs depict the number of PI+ cells in each field for the indicated treatment groups.

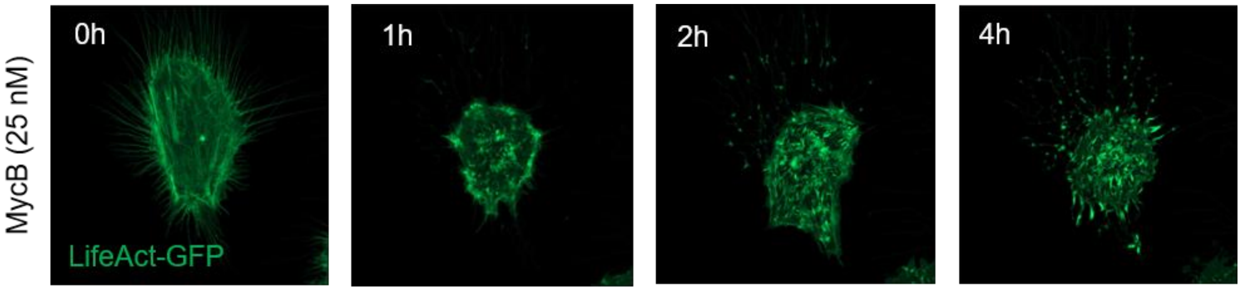

Suppl. Fig. 2. Kinetics of F-actin disruption by Myc B in live SKOV3-LifeAct cells. SKOV3 cells expressing LifeAct-GFP (green) were seeded on live cell chamber coverslips for 24 hours before being treated with either 25 nM of Myc B. Images captured every 1 minute on the LSM 880 Laser Scanning Microscope with Airyscan (Zeiss). Representative confocal live-cell microscopy images are shown for the indicated times following Myc B treatment.

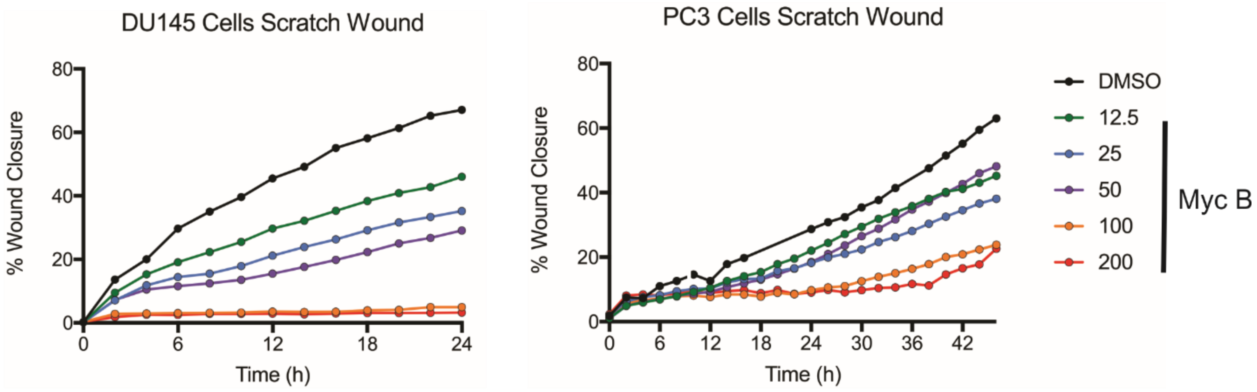

Suppl. Fig 3. Myc B treatment suppresses HER2-negative cancer cell motility. DU145 and PC3 cells (2.5 x10^4) were seeded in triplicate in a 96-well ImageLock plate, and when >90% confluent scratch wounds were produced using the IncuCyte Woundmaker (a 96-well wound making tool). Following wounding, and removal of non-adherent cells, media was added containing DMSO (1%) or Myc B (12.5 – 200 nM) and placed into the IncuCyte Zoom System and analyzed every 2 hours for 24 hours or 48 hours. Graphs depict the percent change in wound area confluence over time of the indicated treatments to measure cell migration (IncuCyte ZOOM Scratch Wound Analysis).

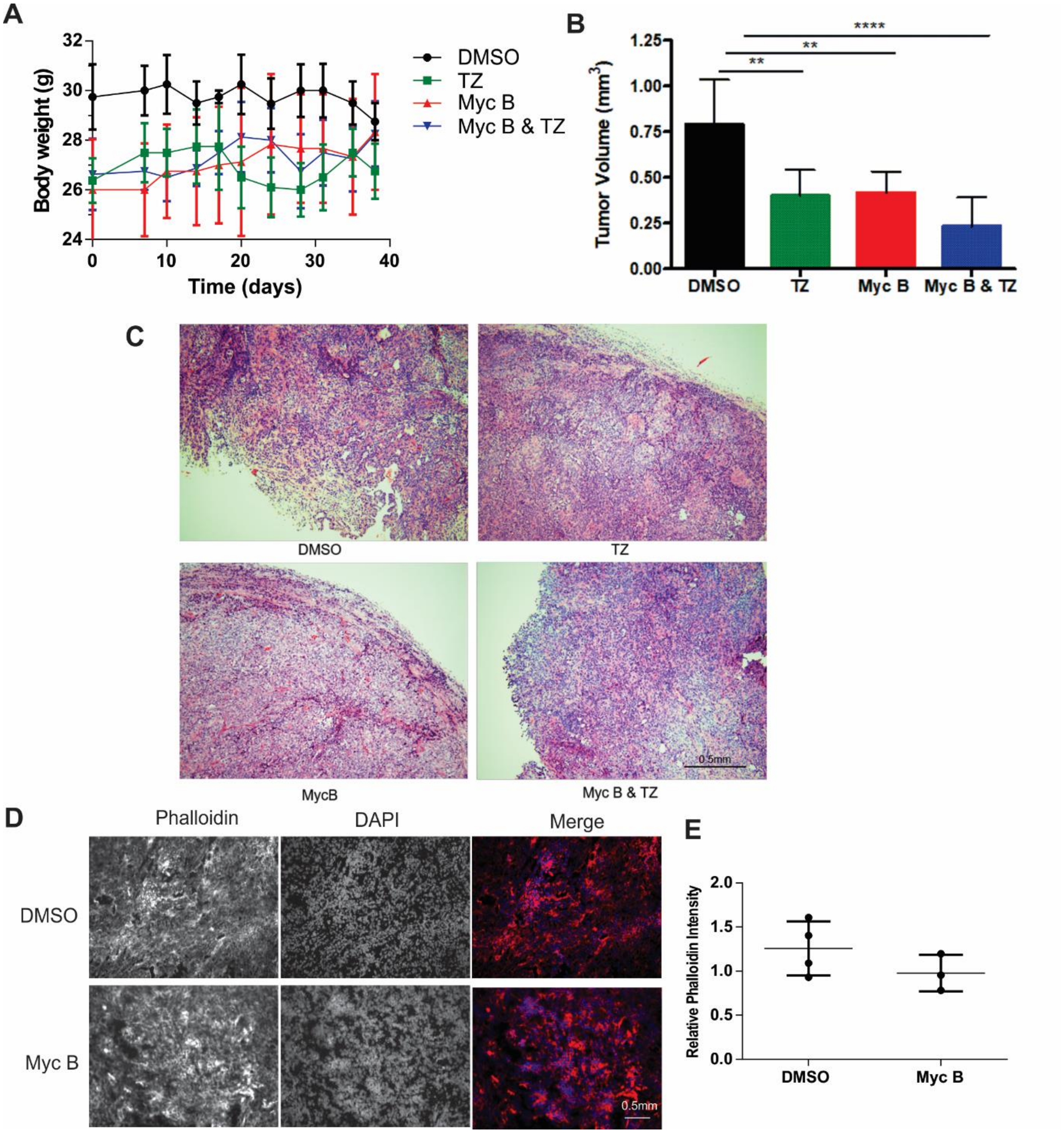

Suppl. Fig. 4. Representative phenotype of SKOV3 subcutaneous xenograft tumor sections. (A) Graph depicts the mean body weights for each treatment group throughout the subcutaneous tumor xenograft study (n=4/group, mean ± SEM). (**B**) Graph depicts mean tumor volume for each treatment group at endpoint (n=6/group; mean ± SEM, ** *P* <0.01, *** *P* <0.001). (**C**) Representative histology images for H&E staining of SKOV3 subcutaneous xenograft tumor sections. **(D)** Cryosections of SKOV3 subcutaneous xenograft tumors from the DMSO and Myc B treatment groups were stained with DAPI and Alexa 555-Phalloidin. A representative epiflourescence micrograph with overlay of both channels is provided for both treatment groups. (**E**) Graph depicts quantification of relative Phalloidin signal intensity relative to that of DAPI for each field, with average value shown for each mouse in DMSO or Myc B treatment groups (n = 3-4/group, mean ± SD).

Supplementary Videos

Supplementary Video 1. Live cell imaging of SKOV3-LifeAct-GFP cells (green) treated with Myc B (25 nM) for up to 6 hours using an LSM 880 Laser Scanning Microscope with Airyscan (Zeiss) equipped with a 60X objective and climate controlled chamber.

Supplementary Video 2. Live cell imaging of SKOV3-LifeAct-GFP cells (green) treated with Myc B (50 nM) for up to 132 minutes using a spinning disk confocal microscope equipped with a 60X objective and climate controlled chamber.

Supplementary Video 3. Live cell imaging of SKOV3-LifeAct-GFP cells (green) treated with DMSO (1%) for up to 137 minutes using a spinning disk confocal microscope equipped with a 60X objective and climate controlled chamber.

Supplementary Video 4. Time lapse video of SKOV3 cell invasion assays upon treatment with DMSO (1%) for 24 hours using the IncuCyte ZOOM system, as described in Methods.

Supplementary Video 5. Time lapse video of SKOV3 cell invasion assays upon treatment with Myc B (25 nM) for 24 hours using the IncuCyte ZOOM system, as described in Methods.
